# Refractive status and optical components in premature infants with and without retinopathy of prematurity: A 4- to 5-year cohort study

**DOI:** 10.3389/fped.2022.922303

**Published:** 2022-11-17

**Authors:** Xuanxuan Xie, Yang Wang, Rulian Zhao, Jing Yang, Xiaohui Zhu, Lijuan Ouyang, Ming Liu, Xinke Chen, Ning Ke, Yong Li, Lianhong Pi

**Affiliations:** ^1^ Department of Ophthalmology, Children's Hospital of Chongqing Medical Universitys, Chongqing, China; ^2^ National Clinical Research Center for Child Health and Disorders, Chongqing, China; ^3^ Ministry of Education Key Laboratory of Child Development and Disorders, Chongqing, China

**Keywords:** premature infants, retinopathy of prematurity, optical components, refractive status, myopia

## Abstract

This study was aimed to investigate the characteristics of refractive parameters in premature infants and children aged 3–8 years with mild retinopathy of prematurity (ROP) and to explore the effects of premature delivery and mild ROP on the development of refractive status and ocular optical components. Premature infants who underwent ocular fundus oculi screening in our hospital between January 2009 and February 2011 were included and divided into the ROP group and the non-ROP group. Full-term infants were the controls. The results of the annual ocular examination conducted between 2014 and 2018 were analysed, and the refractive status, optical components, and developmental trends were compared among the three groups. The total follow-up time was 4–5 years. The prevalence of myopia and astigmatism was high in the ROP group (*P* < 0.05). In the non-ROP group, the prevalence of myopia was also higher than that in the control group. The prevalence of myopia increased with age in the ROP and non-ROP groups, while the prevalence of astigmatism remained unchanged. In the ROP group, the corneal refractive power was the largest, the lens was the thickest and the ocular axis was the shortest; in the control group, the corneal refractive power was the smallest, the lens was the thinnest, and the ocular axis was the longest. These parameters in the non-ROP group were between those in the two groups mentioned above (*P* < 0.05). The corneal refractive power was relatively stable at 3–8 years old in the three groups. The change in lens thickness was small in both the ROP group and the non-ROP group (*P* = 0.75, *P* = 0.06), and the lens became thinner in the control group (*P* < 0.001). The length of the ocular axis increased in the three groups. Preterm infants are more likely to develop myopia than full-term infants, and children with ROP are more likely to develop both myopia and astigmatism. Thicker lenses were the main cause of the high prevalence of myopia in premature infants with or without ROP.

## Introduction

Foetal retinal vasculature mainly develops in the third trimester. Premature delivery can lead to retinal vascular ischaemia, hypoxia, and hypoplasia, resulting in retinopathy of prematurity (ROP) (1). The survival rate of premature babies worldwide has increased over the years (1, 2), and the prevalence of ROP is also increasing (3). ROP is a main cause of blindness in children worldwide and is a controllable and progressive disease (4). Early screening and treatment of ROP in premature infants can prevent the occurrence and progression of ROP and play a crucial role in protecting children's vision. Currently, the screening for and follow-up of ROP in premature infants are aimed to prevent serious complications such as retinal detachment, retinal folds, and an ectopic macula (1, 5). In addition to the serious eye diseases mentioned above, ROP of different severities can also adversely affect the development of various optical components of the eyes and have a long-term impact on refractive status, leading to an increase in the prevalence of myopia and astigmatism in children with ROP (4, 6). It has been reported that the prevalence of myopia is directly proportional to the severity of ROP (7–9). ROP can increase the prevalence of refractive error. In addition, previous studies on the refractive status of preterm infants without ROP have also revealed that the prevalence of myopia was also greater than that in full-term infants. In these studies, researchers report the status of refractive error and optical components in preterm infants and have indicated that premature delivery and ROP can affect refractive status (10).

Currently, many studies on the refractive status and optical components in preterm infants and ROP children are mostly cross-sectional, and children with the disease at the threshold stage are the main subjects (1, 11). There is a lack of long-term, dynamic follow-up of these patients. Moreover, the pathogenesis of myopia is not yet clear in children with ROP. We conducted a 4- to 5-year follow-up of children with mild ROP and preterm infants, and the refractive status and optical components of these subjects were analysed, aiming to explore the long-term effects of mild ROP and premature birth on refractive status and optical components.

## Subjects and methods

Premature infants who underwent ocular fundus screening at the Children's Hospital of Chongqing Medical University between January 2009 and February 2011 were divided into two groups: the ROP group and the non-ROP group. In addition, full-term infants of the same age comprised the control group (gestational age ≥37 weeks or birth weight ≥2,500 g). Subjects had no organic eye diseases (except for ametropia) or family history of severe myopia. We obtained the annual ophthalmologic examination results of patients ages 3–4 to age 8, with a total follow-up of 4–5 years.

### Screening for ROP

The first examination was performed at 4–6 weeks after birth or the corrected gestational age of 32 weeks. Staging and partitioning were conducted according to the 1984 diagnostic criteria for ROP (12), and the follow-up was conducted as follows (13): Zone II, stage 1 or 2 lesions without plus lesions, and Zone III, stage 1 or 2 lesions, examination was performed once weekly; for subjects with prethreshold lesions, the ocular fundus was closely monitored once every 2–3 days; for subjects with threshold lesions, laser therapy or cryotherapy was performed within 72 h; for subjects with stage 4 and 5 lesions, surgery was performed. For subjects who had no ROP and no complete vascularization of the peripheral retina, follow-up was performed once every 2–3 weeks until the retina was completely vascularized.

### Ophthalmologic examination

All the subjects underwent the first ophthalmological examination at 3 or 4 years old and then underwent an annual ophthalmological examination until 8 years old. The corneal refractive power, corneal curvature, anterior chamber depth (ACD), lens thickness (LT), vitreous thickness (VITR), and ocular axial length (AL) were examined, and retinoscopy was performed under ciliary muscle paralysis.

An autorefractor (autorefractor RK-8100; Topcon, Tokyo, Japan) was used to examine corneal refractive power and corneal curvature. They were measured 3 times, and the mean was calculated.

ACD, LT, VITR and AL were measured with an ocular type A ultrasound instrument (KANGH CAS-2000, China). Measurements were performed 8 times, and the mean was calculated.

Retinoscopy under ciliary muscle paralysis: 1% cyclopentolate eye drops were administered 3–4 times. The absence of pupillary light reflex was indicative of complete cycloplegia. Then, retinoscopy was performed using an ophthalmoscope (YZ24; Six Vision Corp., Suzhou, China) (14, 15).

### Data collection and processing

All examinations were performed by specific clinicians. Automatic optometry, eye ultrasonography, drug administration, retinoscopy and data processing were independently performed by 5 clinicians, and each examination was performed by the same clinician in this study. The “triple blind” principle was employed for the subjects, examiner and analyser. The diopter is expressed as the equivalent spherical refraction (SE): SE = spherical refraction + 1/2 cylinder refraction. The distribution of refractive power is expressed as the average refractive power (X) ± standard deviation (SD). Hyperopia was defined as a SE ≥  + 2.00 D, myopia as a SE ≤ −0.50 D, and astigmatism as an absolute cylinder refraction ≥ 1.00 DC (16). The results of each eye were recorded for further analysis.

### Statistical analysis

Continuous variables are expressed as the mean ± standard deviation, and categorical variables are expressed as frequencies or percentages. Statistical analysis was performed with SPSS version 26.0. The prevalence of myopia, hyperopia, and astigmatism was compared using the Chi square test or the Fisher's exact test when more than 20% of the theoretical frequency was <5. If a significant difference was observed among the groups, the rates were compared 10 times for subjects of different ages and 3 times for subjects of the same age (*α* = 0.05/10 = 0.005, *α* = 0.05/3 = 0.0167).

The means of continuous variables among subjects with the same age in the three groups and in subjects at different ages in each group were compared with one-way analysis of variance. A value of *P* < 0.05 was considered statistically significant. When a significant difference was noted among the groups, comparisons were performed with the LSD test if homogeneity of variance was present or Tamhane's T2 test if heterogeneity of variance was present.

## Results

### General characteristics

The first follow-up was performed at 3–4 years old. All subjects underwent an ophthalmological examination and had complete information; they had no organic eye diseases other than ROP, no central nervous or circulatory system diseases, and no family history of severe myopia. ROP patients receiving an intravitreal injection or undergoing laser treatment were also excluded. A total of 132 children were enrolled in the present study. There were 31 subjects in the ROP group, 59 in the non-ROP group, and 42 in the control group. Among the 31 patients with ROP, stage 1 ROP was noted in 17 subjects, stage 2 ROP in 4 subjects, stage 3 ROP in 1 patient and prethreshold stage ROP in 9 subjects, and none had threshold lesions. Among them, 28 patients suffered from ROP in both eyes, and 3 had monocular ROP (1 in the right eye and 2 in the left eye). A total of 132 subjects were followed up for more than 4 years, with an average follow-up time of 4.07 ± 0.92 years. Twenty subjects (7 in the ROP group, 4 in the non-ROP group, and 9 in the control group) were lost to follow-up or had incomplete information. The characteristics of the subjects in each group are shown in Table 1.

**Table 1:** Characteristics of subjects in 3 groups.

	ROP group	non-ROP group	Control group
Number of eyes	59 (31)	118 (59)	84 (42)
Sex (female, %)	61.3 (38.7)	47.5 (52.5)	38.1 (61.9)
GA (mean, weeks)^a^	29.71 ± 0.33	31.59 ± 0.24	39.17 ± 0.29
BW (mean, g)^a^	1,444.36 ± 63.98	1,645.68 ± 46.38	3,313.43 ± 54.96

GA gestational age at birth, BW birth weight.

^a^ *P* < 0.05 (one-way analysis of variance, least-significant difference).

According to the age at follow-up, subjects in each group were divided into 5 subgroups. In the 3–4 years old group, 261 eyes were followed up; in the 5 years old group, 237 eyes were followed up; in the 6 years old group, 245 eyes were followed up; in the 7 years old group, 252 eyes were followed up; and in the 7 years old group, 222 eyes were followed up.

### Prevalence of ametropia

Our study was focused on the prevalence of myopia and astigmatism (Table 2). The prevalence of myopia and astigmatism was significantly different among the three groups (*P* < 0.05). Further analysis showed that only 3 eyes with ROP had myopia in the age 3–4 year group. The prevalence of myopia at 5–8 years old in the ROP group and the non-ROP group was markedly higher than that in the control group (*P* < 0.0167), but there was no significant difference between the ROP group and the non-ROP group. The prevalence of myopia increased with age in both the ROP group and the non-ROP group, but the prevalence of myopia remained relatively stable (*P* = 0.107) in the control group. In the ROP group, the prevalence of myopia remained stable at 3–7 years old, but it increased significantly at 8 years old (*P* < 0.005). In the non-ROP group, the prevalence of myopia remained stable at 3–6 years old, but it increased dramatically at age 7. The increase was the most obvious at age 8, which suggests that the increase in the prevalence of myopia occurred earlier than that in the ROP group. The prevalence of astigmatism was higher in the ROP group than in the non-ROP group (*P* < 0.0167) or the control group, and there was no significant difference between the non-ROP group and the control group (*P* > 0.05). There was a marked difference in the prevalence of astigmatism among the three groups. However, the prevalence of astigmatism remained stable with age. The prevalence of hyperopia decreased with age in the same group (*P* < 0.05), and there was no significant difference in the prevalence of hyperopia among the three groups (*P* > 0.05). Thus, further analysis was not performed.

**Table 2:** Prevalence of myopia and astigmatism in subjects aged 3–8 years.

A. Prevalence of myopia							
Age (years)	ROP group		non-ROP group		Control group		
	Myopia *n* (%)	Total *n*	Myopia *n* (%)	Total *n*	Myopia *n* (%)	Total *n*	*P*
3–4	3 (5.08)	59	0 (0)	84	0 (0)	84	/
5	8 (13.56)	59	5 (5.32)	94	1 (1.19)	84	**0** **.** **008**
6	8 (14.20)	56	7 (6.73)	104	2 (2.22)	90	**0**.**021**
7	9 (18.00)	50	11 (10.51)	110	6 (6.52)	92	**0**.**015**
8	13 (27.66)	47	20 (18.18)	110	5 (7.69)	65	**0**.**02**
*P*	0.028		<0.001		0.107		
B. Prevalence of astigmatism							
Age (years)	ROP group		non-ROP group		Control group		
	Astigmatism *n* (%)	Total *n*	Astigmatism *n* (%)	Total *n*	Astigmatism *n* (%)	Total *n*	*P*
3–4	22 (37.30)	59	25 (28.81)	84	15 (17.86)	84	**0** **.** **028**
5	24 (40.68)	59	17 (18.09)	94	7 (8.33)	84	**<0**.**001**
6	24 (42.85)	56	19 (18.27)	104	8 (8.89)	90	**<0**.**001**
7	18 (36.00)	50	25 (22.73)	110	12 (13.04)	92	**0**.**007**
8	20 (42.55)	47	35 (31.82)	110	12 (18.46)	65	**0**.**018**
*P*	0.932		0.063		0.168		

*P* < 0.05 (one-way analysis of variance; least significant difference test). *n* = number of eyes, ROP = retinopathy of prematurity; categorical data are presented as *n* (%).

### Se and optical components in the three groups

**Figure 1:**
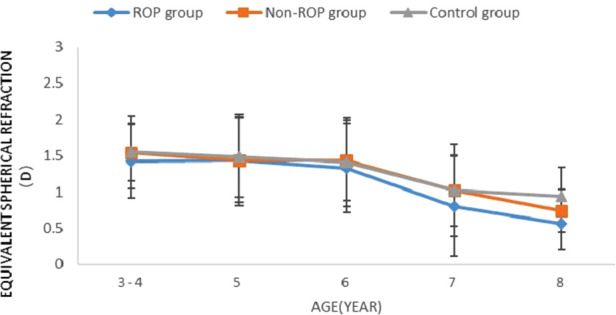
Change in SE in subjects aged 3-8 years. ROP = retinopathy of prematurity; D = dioptres. * Signiﬁcant differences among the study groups (*P* < 0.05, one-way analysis of variance, least significant difference).

Equivalent spherical refraction (SE): There was no significant difference in SE among the ROP group, the non-ROP group and the control group in subjects of the same age (*P* > 0.05). As shown in Figure 1A, SE decreased with age in the three groups (*P* < 0.05). Furthermore, SE remained stable at 3–7 years old (*P* > 0.05), but it decreased dramatically at 8 years old (*P* < 0.05).

Mean corneal refractive power: The mean corneal refractive power (CRP) was the smallest in the control group, followed by the non-ROP group, and was the largest in the ROP group (all *P* < 0.05). In the same group, the mean CRP remained unchanged with age (Table 3, Figure 1A).

**Table 3:** Corneal refractive power, lens thickness, and axial length of subjects at different ages in the three groups.

Optical component	3-4 years (*n* = 261)	5 years (*n* = 237)	6 years (*n* = 245)	7 years (*n* = 252)	8 years (*n* = 222)	*P*
Corneal refractive power (D)						
ROP	43.86 ± 0.78	43.85 ± 0.79	43.87 ± 1.17	44.18 ± 1.21	43.76 ± 2.10	0.075
non-ROP group	43.34 ± 1.62	43.54 ± 1.52	43.55 ± 1.72	43.16 ± 1.7	43.72 ± 1.60	0.062
Control group	43.25 ± 0.17	43.46 ± 1.12	43.44 ± 1.02	42.99 ± 0.74	43.26 ± 1.27	0.177
*P*	**0.014** ^a^	**0.007** ^a^	**0.002** ^a^	**0.002** ^a^	**0.004** ^a^	
Lens thickness (mm)						
ROP	4.47 ± 0.23	4.41 ± 0.21	4.40 ± 0.25	4.47 ± 0.15	4.45 ± 0.22	0.75
non-ROP group	4.44 ± 0.24	4.41 ± 0.25	4.41 ± 0.25	4.45 ± 0.26	4.44 ± 0.28	0.06
Control group	4.40 ± 0.5	4.32 ± 0.27	4.30 ± 0.25	4.03 ± 0.21	3.82 ± 0.15	**<0.001** ^a^
*P*	**0.01** ^a^	**0.031** ^a^	**0.02** ^a^	**0.014** ^a^	**<0.01** ^a^	
Ocular axial length (mm)						
ROP	22.16 ± 0.93	22.32 ± 0.61	22.40 ± 0.98	22.43 ± 0.84	22.51 ± 0.66	**0**.**03**^a^
non-ROP group	22.23 ± 0.78	22.35 ± 0.74	22.40 ± 0.69	22.61 ± 0.8	22.74 ± 1.00	**0**.**02**^a^
Control group	22.30 ± 0.57	22.47 ± 0.56	22.52 ± 0.65	22.78 ± 0.65	22.93 ± 0.57	**<0.001** ^a^
*P*	**0.012** ^a^	**0.008** ^a^	**0.01** ^a^	**0.006** ^a^	**<0.001** ^a^	

^a^ Signiﬁcant differences among the study groups (*P* < 0.05, one-way analysis of variance, least significant difference) ROP = retinopathy of prematurity; mm = millimetre; D = diopter, *n* = number of eyes, Data are expressed as means ± standard deviation.

**Figure 2:**
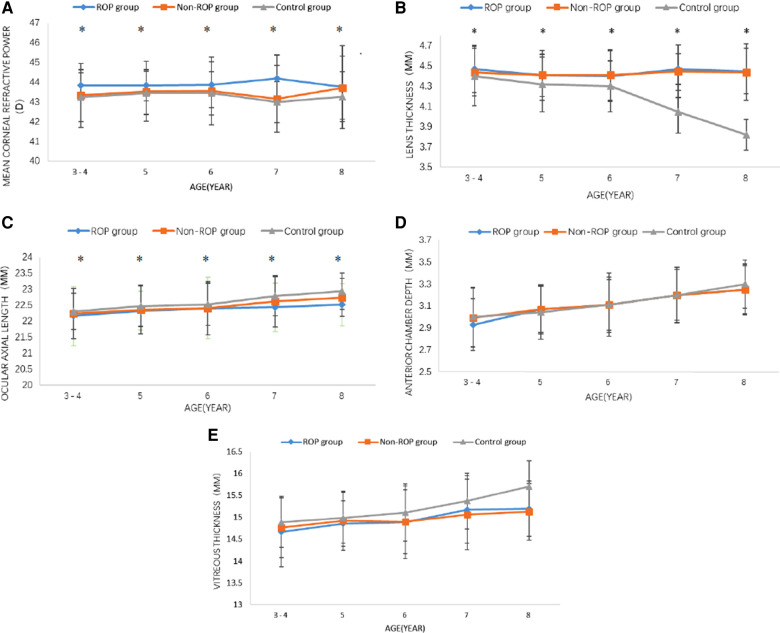
Changes in the optical components at 3–8 years old. (**A**) Corneal refractive power, (**B**) lens thickness, (**C**) ocular axial length, (**D**) anterior chamber depth, (**E**) vitreous thickness. ROP = retinopathy of prematurity; MM = millimetre; D = diopter. *P < 0.05 (one-way analysis of variance, least-significant difference).

Lens thickness (LT): The LT is shown in Figure 2B and Table 3. The results showed that the LT in the non-ROP group and the ROP group was significantly higher than that in the control group, while there was no marked difference in the LT between the ROP group and the non-ROP group (*P* > 0.05), which remained in subjects of different ages. The LT in the control group became thinner with age (*P* < 0.001), then started to decrease at the 5 years old, and remained stable at 6–7 years old, but then decreased significantly at 7–8 years old; in both the ROP and control groups, the LT was not affected by age (*P* > 0.05).

Ocular axial length(AL): As shown in Figure 2C and Table 3, the AL in the control group was longer than that in the non-ROP group and the ROP group (*P* < 0.05). There was no significant difference in the AL between the non-ROP group and the ROP group (*P* > 0.05). The AL increased with age in each group (*P* < 0.05). Further analysis showed that the AL in the control group increased significantly at the 5 years old, and this increase was reduced at 5–6 years old; thereafter, it began to increase dramatically until 7–8 years old (*P* < 0.05). The AL in the non-ROP group and the ROP group remained stable at 3–6 years old (*P* > 0.05) and began to increase at the 7 years old, but there was no significant difference at 7–8 years old (*P* > 0.05).

Anterior chamber depth and vitreous body thickness: There were no significant differences in the anterior chamber depth or vitreous body thickness among the three groups. In the three groups, the mean anterior chamber depth and vitreous body thickness increased with age (*P* < 0.05) (Figures 2D,E).

## Discussion

Advances in perinatal medicine have improved the survival rate of preterm infants worldwide; 15 million infants are born prematurely each year, and postnatal fundus screening to prevent retinopathy of prematurity (ROP) is receiving increasing attention (17). Premature infants are born with immature eye development. Not only ROP, they are also more likely to develop refractive error, amblyopia, strabismus, and other diseases than full-term infants (6, 7). For severe ROP, treatment modalities such as surgery, laser therapy and anti-vascular endothelial growth factor (VEGF) are improving (18). Early screening allowed for an increasing number of mild ROP cases to be detected. Although mild ROP does not require treatment and tends to spontaneously resolve during the infant period, refractive errors are more common in children with mild ROP than in normal children (8, 19). Ophthalmologic care for preterm infants was focused on infancy, aimed at preventing and treating ROP (17). There was a lack of guidance for long-term ophthalmologic follow-up of preterm infants and children with mild ROP. To investigate the effects of prematurity and ROP on ocular development in children, our team collected ophthalmologic data on children with or without ROP and conducted a 4- to 5-year follow-up study.

In this study, the prevalence of myopia in the ROP group and the non-ROP group was higher than that in the control group, which was consistent with previous studies. In some studies, an increased prevalence of myopia in early childhood in children with ROP was found. In the lower age group (age 3–4) of our study, only children with ROP had myopia (5.08%), while the remaining two groups of children had no myopia, which was higher than the 2.28% prevalence of myopia in children of the same age reported by Wang J (20). Thus, it is considered that ROP could lead to an increased risk of myopia in young children, and previous study by Dikopf MS (1) showed that the severity of ROP, the area of the lesion, and receiving ocular treatment are risk factors. During the follow-up, the prevalence of myopia increased in the ROP and non-ROP groups, with 7.69% in the control group, 27.66% in the ROP group, and 18.18% in the preterm group at the last follow-up (age 8). There was no significant difference between the ROP and non-ROP groups (*P* > 0.05). In comparison to Zhihao Xie's study (21), the prevalence of myopia among the 8-year-old general population in the same region was 9.6%, which was similar to the prevalence in the control group in our study and lower than that in the ROP and non-ROP groups. The prevalence of myopia in premature children with or without mild ROP in the study by Wang J (7) was significantly higher than that of full-term children, 11% at 7 years old, which was close to the total prevalence of the ROP group and the non-ROP group in our study, which was 12.50% at age 7. It was speculated that not only ROP but also prematurity leads to an increased risk of myopia, which was in line with the findings of a study of preterm infants without ROP conducted by Hsieh CJ (22). Their study also showed that low gestational age and birth weight led to an increased risk of myopia.

We had also done an analysis on astigmatism. The prevalence of astigmatism in the ROP group in our study was higher than that in the non-ROP and control groups. Davitt BV (3, 23) reported that astigmatism formation in children with ROP occurred in early childhood and that laser treatment had a large impact. Compared with the same age group in his study, the prevalence of astigmatism in our study was lower, which might be because the ROP group in our study did not undergo laser photocoagulation. It was considered that, in addition to retinal scarring caused by laser treatment, mild ROP may also lead to an increased prevalence of astigmatism, which is in line with the previous studies (7, 8, 24). During orthokeratology in children, the cornea has a large refractive power and reaches a stable corneal refractive power at age 2 (25, 26). The corneal refractive power (CRP) was stable during the follow-up of our study and was always greatest in the ROP group. The greater the CRP, the steeper the cornea. There was no significant change in the prevalence of astigmatism with increasing age, which was synchronous with the change in CRP, considering that the high incidence of astigmatism in patients with ROP was related to corneal development.

Meanwhile, the refractive status and optical components of these subjects were analysed in our study. There was no statistically difference in spherical refraction (SE) among the three groups, with a significant decrease at the last follow-up (age 8), similar to the findings of a study by Wang (27). In their study, children aged 7–8 years from the same region showed a decrease in SE compared to children aged 3–6 years, which was considered to be related to the progression of emmetropization. In the normal population, the increase in the axial length (AL) was an important reason for the increase in the prevalence of myopia (25, 26). But in our study, the ROP and non-ROP groups have large myopia prevalence with low AL. Which made us think that AL was not the main reason for the high prevalence of myopia or its increased prevalence in the non-ROP and ROP groups. The AL in each group increased significantly (*P* < 0.05) at the last examination compared to preschool age (3–6 years), which was similar to the study by Mutti (25), which was considered to be related to the decrease in outdoor activities with sufficient light (28, 29). CRP, lens thickness (LT), and AL are determinants of ocular refractive power (26, 30). After 5 years of follow-up, we concluded that the preterm and ROP groups had thick lenses, steep corneas, and short eye axes, which was consistent with those found in previous studies (8, 11, 31). The decrease in LT in normal children was synchronized with the increase in the AL, thus compensating for the refractive changes associated with the increase in AL. The thinner the lens, the lower the refractive power of the lens. Each +1.00 D reduction in lens refractive power compensated for a 0.33 mm increase in AL and buffers −1.00 of myopia (32). If the increase in the AL exceeded the compensation of the lens, it led to the onset of myopia (25, 33). LT decreased with age only in the control group, and the increase in myopia prevalence was considered to be attributed to an imbalance in the development of LT and AL.

The cause of increased LT in ROP and preterm children was unknown. Previous study (11) showed that ROP and prematurity lead to blocked development of the anterior segment of the eye, which affected the process of orthokeratology and refractive status. Another theory was the neuroectodermal hypothesis, which refers to the absence of an anterior segment block leading to an increase in LT due to altered neuroectodermal development in patients with ROP. It has been suggested that ROP lesions are located at the site of the eye with the fastest growth, which in turn limits the growth of the anterior sclera and the preoptic segment (34). Additionally, there was a theory that inadequate temperature or biological stress during corneal growth and development could also lead to developmental blockage of the anterior segment. This developmental abnormality could adversely affect the development of the entire eye (35, 36). In future studies, a visual electrophysiological examination is needed in children with ROP to analyse retinal development in preterm infants in childhood.

In summary, both prematurity and ROP can lead to an increased prevalence of myopia, and ROP can lead to an increased prevalence of astigmatism. In the ROP group and the non-ROP group, children had greater corneal refractive power, thicker lenses, and shorter ocular axes. The larger LT mismatch with the development of the ocular axis may explain the high prevalence of myopia in the children in the two groups mentioned above. A separate analysis of optical components in children with refractive error was lacking in our study and could be added in future studies. For premature infants with or without ROP, a long-term follow-up of the eyes is needed. If refractive error is present, early intervention and correction are warranted to improve long-term visual quality and quality of life.

## Data Availability

The raw data supporting the conclusions of this article will be made available by the authors, without undue reservation.

## References

[1] Dikopf MS Machen LA Hallak JA Chau FY Kassem IS. Zone of retinal vascularization and refractive error in premature eyes with and without spontaneously regressed retinopathy of prematurity. J AAPOS. (2019) 23(4):211.e1–e6. 10.1016/j.jaapos.2019.03.006 PMC6778011 31229607

[2] Edy Siswanto J Sauer PJ. Retinopathy of prematurity in Indonesia: incidence and risk factors. J Neonatal Perinatal Med. (2017) 10(1):85–90. 10.3233/NPM-915142 28304327

[3] Davitt BV Quinn GE Wallace DK Dobson V Hardy RJ Tung B Astigmatism progression in the early treatment for retinopathy of prematurity study to 6 years of age. Ophthalmology. (2011) 118(12):2326–9. 10.1016/j.ophtha.2011.06.006 21872933 PMC3227788

[4] Deng Y Yu CH Ma YT Yang Y Peng XW Liao YJ Analysis of the clinical characteristics and refraction state in premature infants: a 10-year retrospective analysis. Int J Ophthalmol. (2019) 12(4):621–6. 10.18240/ijo.2019.04.16 31024817 PMC6469563

[5] Kim SJ Port AD Swan R Campbell JP Chan R Chiang MF. Retinopathy of prematurity: a review of risk factors and their clinical significance. Surv Ophthalmol. (2018) 63(5):618–37. 10.1016/j.survophthal.2018.04.002 29679617 PMC6089661

[6] Petriçli İS Kara C Arman A. Is being small for gestational age a risk factor for strabismus and refractive errors at 3 years of age. Turk J Pediatr. (2020) 62(6):1049–57. 10.24953/turkjped.2020.06.017 33372444

[7] Wang J Ren X Shen L Yanni SE Leffler JN Birch EE. Development of refractive error in individual children with regressed retinopathy of prematurity. Invest Ophthalmol Vis Sci. (2013) 54(9):6018–24. 10.1167/iovs.13-11765 23920368 PMC3771557

[8] Kaya M Berk AT Yaman A. Long-term evaluation of refractive changes in eyes of preterm children: a 6-year follow-up study. Int Ophthalmol. (2018) 38(4):1681–8. 10.1007/s10792-017-0642-z 28669100

[9] Korkmaz L Karaca C Akin MA Bastug O Sahiner M Ozdemir A Short-term refractive effects of propranolol hydrochloride prophylaxis on retinopathy of prematurity in very preterm newborns. Curr Eye Res. (2018) 43(2):213–7. 10.1080/02713683.2017.1390769 29135357

[10] Ozdemir O Tunay ZO Acar DE Acar U. Refractive errors and refractive development in premature infants. J Fr Ophtalmol. (2015) 38(10):934–40. 10.1016/j.jfo.2015.07.006 26542677

[11] Chang S Lee YS Wu SC See LC Chung CC Yang ML Anterior chamber angle and anterior segment structure of eyes in children with early stages of retinopathy of prematurity. Am J Ophthalmol. (2017) 179:46–54. 10.1016/j.ajo.2017.04.010 28450043

[12] An international classification of retinopathy of prematurity. The committee for the classification of retinopathy of prematurity. Arch Ophthalmol. (1984) 102(8):1130–4. 10.1001/archopht.1984.01040030908011 6547831

[13] Fierson WM. Screening examination of premature infants for retinopathy of prematurity. Pediatrics. (2018) 142(6). 10.1542/peds.2018-3061. www.aappublications.org/news 30478242

[14] French AN Morgan IG Burlutsky G Mitchell P Rose KA. Prevalence and 5- to 6-year incidence and progression of myopia and hyperopia in Australian schoolchildren. Ophthalmology. (2013) 120(7):1482–91. 10.1016/j.ophtha.2012.12.018 23522969

[15] Yazdani N Sadeghi R Momeni-Moghaddam H Zarifmahmoudi L Ehsaei A. Comparison of cyclopentolate versus tropicamide cycloplegia: a systematic review and meta-analysis. J Optom. (2018) 11(3):135–43. 10.1016/j.optom.2017.09.001 29132914 PMC6039578

[16] Zhu X Zhao R Wang Y Yang W Ouyang L Yang J Refractive state and optical compositions of preterm children with and without retinopathy of prematurity in the first 6 years of life. Medicine (Baltimore). (2017) 96(45):e8565. 10.1097/MD.0000000000008565 29137074 PMC5690767

[17] Adams G. ROP In Asia. Eye (Lond). (2020) 34(4):607–8. 10.1038/s41433-019-0620-y 31582794 PMC7265387

[18] Chan-Ling T Gole GA Quinn GE Adamson SJ Darlow BA. Pathophysiology, screening and treatment of ROP: a multi-disciplinary perspective. Prog Retin Eye Res. (2018) 62:77–119. 10.1016/j.preteyeres.2017.09.002 28958885

[19] Lue CL Hansen RM Reisner DS Findl O Petersen RA Fulton AB. The course of myopia in children with mild retinopathy of prematurity. Vision Res. (1995) 35(9):1329–35. 10.1016/0042-6989(94)00227-D 7610594

[20] Wang J Liu J Ma W Zhang Q Li R He X Prevalence of myopia in 3-14-year-old Chinese children: a school-based cross-sectional study in Chengdu. BMC Ophthalmol. (2021) 21(1):318. 10.1186/s12886-021-02071-6 34470605 PMC8411514

[21] Xie Z Long Y Wang J Li Q Zhang Q. Prevalence of myopia and associated risk factors among primary students in Chongqing: multilevel modeling. BMC Ophthalmol. (2020) 20(1):146. 10.1186/s12886-020-01410-3 32295555 PMC7161106

[22] Hsieh CJ Liu JW Huang JS Lin KC. Refractive outcome of premature infants with or without retinopathy of prematurity at 2 years of age: a prospective controlled cohort study. Kaohsiung J Med Sci. (2012) 28(4):204–11. 10.1016/j.kjms.2011.10.010 22453068 PMC11916860

[23] Davitt BV Dobson V Quinn GE Hardy RJ Tung B Good WV. Astigmatism in the early treatment for retinopathy of prematurity study: findings to 3 years of age. Ophthalmology. (2009) 116(2):332–9. 10.1016/j.ophtha.2008.09.035 19091409 PMC2692212

[24] Pétursdóttir D Holmström G Larsson E. Refraction and its development in young adults born prematurely and screened for retinopathy of prematurity. Acta Ophthalmol. (2022) 100(2):189–95. 10.1111/aos.14766 33528099

[25] Mutti DO Mitchell GL Jones LA Friedman NE Frane SL Lin WK Axial growth and changes in lenticular and corneal power during emmetropization in infants. Invest Ophthalmol Vis Sci. (2005) 46(9):3074–80. 10.1167/iovs.04-1040 16123404

[26] Guo X Fu M Ding X Morgan IG Zeng Y He M. Significant axial elongation with minimal change in refraction in 3- to 6-year-old Chinese preschoolers: the shenzhen kindergarten eye study. Ophthalmology. (2017) 124(12):1826–38. 10.1016/j.ophtha.2017.05.030 28711218

[27] Yang Wang RZ Pi L. Relationship between axial length, lens power, and refractive error in. Chin J Optom Ophthalmol Vis Sci. (2020) 22(03):191–7.

[28] Jiang X Kurihara T Torii H Tsubota K. Progress and control of myopia by light environments. Eye Contact Lens. (2018) 44(5):273–8. 10.1097/ICL.0000000000000548 30048342

[29] Wu PC Chen CT Lin KK Sun CC Kuo CN Huang HM Myopia prevention and outdoor light intensity in a school-based cluster randomized trial. Ophthalmology. (2018) 125(8):1239–50. 10.1016/j.ophtha.2017.12.011 29371008

[30] Tideman JW Snabel MC Tedja MS Rijn GA Wong KT Kuijpers RWAM Association of axial length with risk of uncorrectable visual impairment for europeans with myopia. JAMA Ophthalmol. (2016) 134(12):1355–63. 10.1001/jamaophthalmol.2016.4009 27768171

[31] Özdemir Ö Özen Tunay Z Ergintürk Acar D. Growth of biometric components and development of refractive errors in premature infants with or without retinopathy of prematurity. Turk J Med Sci. (2016) 46(2):468–73. 10.3906/sag-1501-40 27511513

[32] Mutti DO Sinnott LT Lynn Mitchell G Jordan LA Friedman NE Frane SL Ocular component development during infancy and early childhood. Optom Vis Sci. (2018) 95(11):976–85. 10.1097/OPX.0000000000001296 30339640 PMC6212316

[33] Iribarren R. Crystalline lens and refractive development. Prog Retin Eye Res. (2015) 47:86–106. 10.1016/j.preteyeres.2015.02.002 25683786

[34] Beri S Malhotra M Dhawan A Garg R Jain R D'souza P. A neuroectodermal hypothesis of the cause and relationship of myopia in retinopathy of prematurity. J Pediatr Ophthalmol Strabismus. (2009) 46(3):146–50. 10.3928/01913913-20090505-05 19496495

[35] Li ML Hsu SM Chang YS Shih MH Lin YC Lin CH Retinopathy of prematurity in southern Taiwan: a 10-year tertiary medical center study. J Formos Med Assoc. (2013) 112(8):445–53. 10.1016/j.jfma.2012.03.002 24016609

[36] Fielder AR Quinn GE. Myopia of prematurity: nature, nurture, or disease. Br J Ophthalmol. (1997) 81(1):2–3. 10.1136/bjo.81.1.2 9135397 PMC1722011

